# Argentina’s most important contributions in the field of electrophysiology

**DOI:** 10.1016/j.hroo.2023.10.005

**Published:** 2023-10-26

**Authors:** Marcelo V. Elizari, Luis Aguinaga

**Affiliations:** ∗ Academia Nacional de Medicina, Buenos Aires, Argentina; † Centro Integral de Arritmias Tucumán, Tucumán, Argentina

**Keywords:** Electrocardiology, Contributions, Argentina, Electrophysiology

## Abstract

Latin American electrocardiology emerged internationally thanks to the Argentine School of Electrocardiology. All started when the idea of a different anatomy of the conduction system was not only necessary to change the paradigm of a bifascicular system, but also to question diagnostic electrocardiographic criteria adopted by the scientific community without dispute. Almost every scientific contribution coming from the Argentine School of Electrocardiology represented a significant step forward in the understanding of the electrophysiology of the heart and its electrocardiographic counterpart. There is another reason that increases their value: the noticeable simplicity of the technical facilities with which these studies were done from the modest laboratory in Argentina, whose production was purely and genuinely Latin American. In the following lines we summarize what we consider to be the greatest contributions of the Argentine school to world electrophysiology.

After the Second World War, both clinical and basic cardiologic research found unexpected opportunities in Europe and America, with remarkable contributions to electrocardiography, cellular electrophysiology, and cardiac clinical electrophysiology. Thus, cardiac electrocardiology emerged as a noninvasive and invasive clinical discipline. In Latin América, 2 schools stood out for their scientific contributions and countless disciples: the Mexican school led by Demetrio Sodi-Pallares and the Argentine one created and conducted by Mauricio B. Rosenbaum.

## The trifascicular distribution of the intraventricular conduction system and the concept of hemiblocks

As from the 60s, Latin American electrocardiology emerged internationally thanks to the Argentine School of Electrocardiology (ASE). It all started when the idea of a different anatomy of the conduction system was necessary not only to change the paradigm of a bifascicular system, but also to question diagnostic electrocardiographic criteria adopted by the scientific community without dispute.

For those years, Mauricio B. Rosenbaum was unable to interpret and explain the electrocardiography (ECG) from a patient, despite being already an excellent, experienced electrocardiographer. This ECG showed totally different and opposing images every day. Changes in the electrical axis from –60° to +120° were so noticeable that he first presumed it was about electrode misplacement, until those changes occurred in the same registered tracing. A couple of years later, he came across another similar ECG. In both cases, the patients had permanent right bundle branch block, but the AQRS shifted from –60° to +120°. Rosenbaum suspected that the enigma was related to changes in the electrical conduction within the left ventricle that followed different conduction pathways, but it was impossible to interpret and analyze given what was known at the moment about the anatomy of the left branch of the conduction system. In fact, the classic anatomic knowledge stated that there were only 2 electrical terminals in the ventricles, one on the right and another on the left, and as such, the system was considered bifascicular. However, this apparently so logical concept overlooked many electrocardiographic signs that had been unattended for decades. In this way, these two ECGs gave birth to the idea that the intraventricular conduction system was not exactly bifascicular. In the 60s, the idea of a different anatomy away from the classic descriptions was sheer heresy because it was necessary not only to change the paradigm of a bifascicular system, but also to question diagnostic electrocardiographic criteria adopted by the scientific community without dispute. Rosenbaum’s reasoning was simple: if the right bundle branch block was constant, the impulse should reach the left ventricle exclusively through the left branch, and then the stimulus should divide into two; if it took one of the pathways, the AQRS shifted upward and to the left at about –60°. Conversely, if it took the other one, it shifted downward and to the right at about +120°. This interpretation led to the important conclusion that in the human ventricle there are 2 different pathways or conductors and that, in pathological or physiological conditions, each of those pathways could be blocked in a separate fashion so that the stimulus could circulate along one or the other. The hypothesis of a different anatomical configuration of the conduction system by systematically studying the cardiac anatomy in several animal species and in humans was tested. The results of 8 years of anatomical, experimental, and clinical investigations were published in the book *Los Hemibloqueos* (*The Hemiblocks*),^1^ and the concept of the trifascicular distribution of the intraventricular conducting system was definitely settled. The description of the trifascicular nature of the intraventricular conduction system helped to identify all types of intraventricular blocks: experimental hemiblocks include the production of divisional left bundle branch block and trifascicular heart block.^1^, ^2^, ^3^ Isolated blocks of the divisions of the left bundle, bifascicular blocks, and trifascicular blocks were then recognized in everyday electrocardiographic reading. Functional or physiological blocks during aberrant intraventricular conduction of premature supraventricular beats constituted invaluable material for the study of the changes that left anterior and left posterior hemiblocks produce on the normal and abnormal ECG.^1^, ^2^, ^3^ The site of origin and mechanism of ventricular wide and narrow extrasystoles were described in detail.

Besides, it also focused on a mechanism of the so-called bidirectional tachycardia, the relationship between conduction disturbances in coronary disease and myocardial infarction or other congenital or acquired pathologies. In conclusion, the existence and recognition of hemiblocks filled an important gap in electrocardiography.

The study of the hemiblocks and the trifascicular conduction system illustrates a clear example of the work philosophy of the ASE: thorough scientific research conducted to solve the unknown posed by clinical observation, acquire a full knowledge on the subject, and form a plausible hypothesis to account for an unrevealed phenomenon and its testing by a scientific method from the basic aspects.

When The Hemiblocks celebrated its 10th anniversary, under the title Historical Milestones, Herman Uhley^4^ stated,

The concept of trifascicular conduction has had a major effect on the contemporary practice of cardiology, influencing the work of clinicians, researchers and students. […] Clinicians gained insight into previously unexplained phenomena and were able to apply these new concepts to the daily interpretation of patients’ electrocardiograms. […] Perhaps the greatest testimony to their contribution is the large number of related publications by many authors since the printing of “Los Hemibloqueos” more than 10 years ago.

Leonard Dreifus stated at the Presidential Citation of the American College of Cardiology in 1975 that

Dr. Rosenbaum and his co-workers’ findings represent a fundamental advance in the understanding of intraventricular conduction disturbance. […] It is particularly important that these observations were brought into sharp focus when so much confusion had existed in the understanding of conduction disturbances.

In the book *The History of Cardiology*, Acierno^5^ stated,

Rosenbaum and co-workers were the prime movers who put together the cumulative experimental and clinical data that led to the creation of a comprehensive concept so that now we think in terms of hemiblocks and trifascicular block. The concept acted as a catalyst for further investigations by anatomists, pathologists and electrophysiologists.

Between 1969 and 1973, more than 30 articles on this breakthrough were published by the Argentine group in renowned international journals.^6^, ^7^, ^8^, ^9^, ^10^ While from 1960 to 1968, no studies on these topics were published, between 1969 and 1974 more than 250 articles about them appeared in cardiology journals, and according to a search in the Citation Index, the articles published by the ASE were cited in more than 3,000 publications in world scientific literature.

Of note, and with reference to these statements, it is worth mentioning that the discovery of fascicular and trifascicular blocks coincided with 2 major technical advances in the field, both in the United States. One was the register of the human His bundle with the pioneering studies by Benjamin Scherlag^11^ and Anthony Damato^12^; the other one was the development of artificial pacemakers. Remarkably, the 3 discoveries, besides being contemporary by chance, were also complementary and altogether facilitated considerable progress on diagnosis and treatment of cardiac conduction disturbances. Indeed, an electrophysiological study could not (and cannot) be interpreted without taking into account the trifascicular nature of the conduction system, and the decision of a pacemaker implant started to depend, in many cases, on applying the concepts and criteria established by the ASE.

## Other scientific contributions

Between 1970 and 1980, the Argentine group conducted and performed a series of original investigations on the physiology and physiopathology of the electrocardiographic abnormalities and manifestations of the specialized system of the heart and arrhythmias, also correlated with in vivo and in vitro experimental models in the laboratory of the Ramos Mejía Hospital. Thus, the experimental production of phase 3 and phase 4 block and of paroxysmal atrioventricular block were correlated with their clinical counterpart.^13^, ^14^, ^15^, ^16^, ^17^ Other studies published were normal and abnormal ventricular automaticity in the human heart, clinical studies on the mechanisms of ventricular arrhythmias, recovery of impulse propagation in the bundle branches of the human heart, a reappraisal of supernormal conduction, the mechanisms of Mobitz II periodicities, the different varieties of 2:1 bundle branch block, the electrophysiologic mechanisms of aberrant ventricular conduction, the making of bundle branch block, physiologic properties of accessory pathways, the mechanism of intermittent bundle branch blocks, and the electrotonic modulation of the T-wave and cardiac memory. All these topics were presented and discussed in a memorable symposium held in Buenos Aires in 1981 and then reported in the multiauthored book *Frontiers of Cardiac Electrophysiology*.^18^

The development of the concept of phase 3 and phase 4 block highlighted an existing relationship between automaticity and conduction. Curiously, intermittent bundle branch block, paroxysmal atrioventricular block, Wolff-Parkinson-White syndrome, and parasystole are all different electrocardiographic conditions that, apparently, are not brought together and whose manifestations seem to differ. Nevertheless, all of them, under certain circumstances, can share a similar underlying electrophysiologic mechanism displaying the occurrence of phase 3 and/or phase 4 block.^19^

## More contributions

Other contributions were the fatigue phenomenon in intraventricular conduction,^20^ normal and pathologic supernormality in the conduction system, and accessory pathways of Wolff-Parkinson-White syndrome.^21^ Original studies on ventricular parasystole were modulation of parasystolic activity by nonparasystolic beats^22^ and concealed ventricular parasystole uncovered in the form of ventricular escapes of variable coupling.^23^ Other interesting contributions were the description of the different forms of Wenckebach block occurring in the bundle branches,^24^ pseudo 2:1 atrioventricular block and T-wave alternans as a manifestation of long QT syndrome, left anterior hemiblock obscuring the diagnosis of right bundle branch block,^25^ electrocardiographic manifestations and electrophysiologic mechanisms of ventricular aberrant conduction of supraventricular premature beats,^1^^,^^3^^,^^18^ etc. The conclusions of these studies may be considered scientific contributions and advances in clinical cardiology, especially effective for the correct interpretation and treatment of arrhythmias.

## Amiodarone

After the discovery of amiodarone as an antiarrhythmic agent, its effects on the treatment of the wide spectrum of cardiac arrhythmias were explored.^26^, ^27^, ^28^ Amiodarone proved to be effective and safe in patients with coronary artery disease, severe congestive heart failure with structural myocardial damage, and any form of cardiac arrhythmias. To highlight, at present, amiodarone is reserved as the last option for the treatment of arrhythmias in patients experiencing severe myocardial structural damage, with or without cardiac failure, coronary artery disease, or ventricular hypertrophies, including electrical storms in the presence of implantable-cardioverter defibrillators, conditions in which the other drugs available in the therapeutic armamentarium are contraindicated.

## Cardiac memory

These were alerted by Chatterjee and colleagues’ observations,^29^ which showed persisting ST-T wave changes after right ventricular pacing. Focusing on this idea, a series of studies were scheduled and it was demonstrated that these ST-T wave alterations occurred in any clinical condition showing a sustained change in ventricular depolarization. Typical examples of this phenomenon are observed in patients with intermittent bundle branch block, ventricular pacing, ventricular premature beats, ventricular tachycardia, and ventricular pre-excitation. Ideally, these changes became apparent after returning to sinus rhythm and normal intraventricular conduction. Given that the abnormal T waves during normal conduction followed and persisted for some time with the same direction of the main forces of the previous abnormal QRS complex, the rather provocative term “cardiac memory” was coined.^18^^,^^30^ Because these T-wave changes perfectly simulate myocardial ischemia, they were called pseudoprimary changes. Interestingly, appropriate ventricular pacing can also normalize chronic abnormal T waves.^31^

## Studies on coronary disease

As from the 80s, the ASE carried out a series of studies and published their results in the electrocardiographic manifestations of coronary artery disease and the antiarrhythmic treatment in patients with acute and chronic syndromes.^32^, ^33^, ^34^

## Research on the pathogenesis of Chagasic cardiomyopathy an idiopathic dilated myocardiopathy

From the early 90s, the Argentine group started working in a very interesting hypothesis dealing with the relationship between anti-β-adrenergic receptors antibodies and anti-M2 cholinergic receptor antibodies and cardiac arrhythmias and their biochemical effects in Chagasic myocarditis and idiopathic dilated cardiomyopathy. Several studies showed that the effect of these antibodies acting against the second extracellular domain of the β-adrenergic receptor are associated with the pathogenesis of ventricular arrhythmias in dilated cardiomyopathy, whereas those acting against M2 cholinergic receptors participate in the pathogenesis of sinus node dysfunction in Chagas heart disease.^35^^,^^36^ Moreover, it was postulated that these antibodies take part in the pathophysiology of the myocardial damage and the development of cardiac failure in patients with dilated cardiomyopathy. In fact, immunoapheresis removing these antibodies from the blood markedly ameliorates symptoms and improves the hemodynamic variables in patients experiencing these pathologies.^37^^,^^38^

## Latest contributions

After the regrettable loss of the leader and founder of the ASE in 2003, more than a hundred articles, books, and chapters in books about diagnosis, physiopathology, and treatment of cardiac arrhythmias were published. Intracellular studies were carried out on the pharmacodynamics of antiarrhythmic drugs.^39^, ^40^, ^41^, ^42^ In vitro studies with microelectrode technique in normal and abnormal human myocardial tissues obtained from explanted hearts were performed.^43^ Two original contributions were related to Brugada syndrome with reference to its etiopathogenesis and diagnosis.^44^^,^^45^ According to Pedro Brugada,^46^ there are at present 3 hypotheses regarding the pathophysiology of the syndrome. He refers to them as the Amsterdam, New York, and Buenos Aires hypotheses. Brugada alludes to the Buenos Aires hypothesis as a fascinating one. Updated studies on cardiac memory and clinical and experimental evidence of supernormal excitability and conduction were published.^47^^,^^48^

In conclusion, almost every scientific contribution coming from the ASE represented a significant step forward in the understanding of the electrophysiology of the heart and its electrocardiographic counterpart. There is another reason that increases their value: the noticeable simplicity of the technical facilities with which these studies were done from the modest laboratory in Argentina, whose production was purely and genuinely Latin American. One of the most remarkable features of the ASE was its critical spirit. This essential principle in science was perfectly defined by José Ingenieros, an Argentine medical doctor and philosopher, who stated,

The progress in art and literature, science and philosophy, moral and politics is always achieved by spirits of rebellion. Those who are domesticated waste their lives along the beaten tracks of thought and action, worshipping idols and shoring up ruins: instead, rebels provide a fruitful and creative work, tirelessly shedding new light upon the trails humankind will walk upon later.^49^
